# Garlic and Onion Attenuates Vascular Inflammation and Oxidative Stress in Fructose-Fed Rats

**DOI:** 10.1155/2011/475216

**Published:** 2011-08-25

**Authors:** Marcela Alejandra Vazquez-Prieto, Cecilia Rodriguez Lanzi, Carina Lembo, Claudio Rómulo Galmarini, Roberto Miguel Miatello

**Affiliations:** ^1^Departamento de Patología, Facultad de Ciencias Médicas, Universidad Nacional de Cuyo, Avenida Libertador 80, 5500 Mendoza, Argentina; ^2^Laboratorio de Fisiopatología Cardiovascular, Instituto de Medicina y Biología Experimental de Cuyo (IMBECU), Consejo Nacional de Investigaciones Científicas y Técnicas (CONICET), Mendoza, Argentina; ^3^Instituto de Horticultura, Facultad de Ciencias Agrarias, Universidad Nacional de Cuyo, EEA INTA La Consulta y CONICET, Mendoza, Argentina

## Abstract

This study evaluates the antioxidant and the anti-inflammatory properties of garlic (G) and onion (O) in fructose-fed rats (FFR). Thirty-day-old male Wistar rats were assigned to control (C), F (10% fructose in drinking water), F+T (tempol 1 mM as control antioxidant), F+G, and F+O. Aqueous G and O extracts were administered orally in doses of 150 and 400 mg/kg/d respectively, and along with tempol, were given during the last 8 weeks of a 14-week period. At the end of the study, FFR had developed insulin resistance, aortic NADPH oxidase activity, increased SBP, plasma TBARS and vascular cell adhesion molecule-1 (VCAM-1) expression in mesenteric arteries, and a decrease in heart endothelial nitric oxide synthase (eNOS). Garlic and onion administration to F rats reduced oxidative stress, increased eNOS activity, and also attenuated VCAM-1 expression. These results provide new evidence showing the anti-inflammatory and antioxidant effect of these vegetables.

## 1. Introduction

Metabolic syndrome (METS), characterized by hypertension, hyperlipidemia, diabetes mellitus, and obesity is an important risk factor for cardiovascular disease [1]. One of the most important causes that contribute to the growing worldwide prevalence of METS, obesity, and type 2 diabetes is the change of dietary habits principally due to the increased intake of simple sugars, mainly fructose, commonly used in the food industry and sugar-sweetened drinks [2]. Feeding carbohydrate-enriched diets to normal rats has been shown to induce insulin resistance and hyperinsulinemia associated with an elevation of systolic blood pressure (SBP) [3]. Fructose-fed rats (FFRs) provide a model of dietary-induced insulin resistance, which has been used to assess the pathophysiological mechanisms of the metabolic and cardiovascular changes associated with METS [4, 5]. Endothelial dysfunction is also associated with this experimental model [6]. Our group previously reported a decrease of the endothelial nitric oxide synthase activity (eNOS) at cardiovascular level, and an increase in oxidative stress and vascular alterations [5, 7].

The major sources of reactive oxygen species (ROS) in vascular tissue are membrane-associated NAD(P)H oxidases [8]. An increase in ROS has been implicated as an important mechanism contributing to endothelial dysfunction and to the pathogenesis of many cardiovascular diseases and their risk factors like METS [9]. ROS induce the expression of different molecules in the endothelial cell surface such as vascular cell adhesion molecule-1 (VCAM-1), which stimulates monocyte binding and subsequent macrophage differentiation [9]. We previously demonstrated that FFR developed vascular remodeling reversed by chronic aspirin administration [10] supporting the hypothesis that vascular inflammation contributes to arterial vascular remodeling [11]. 

There is growing interest in the utilization of herbal and food plants in the prevention of several diseases such as cardiovascular disease [12]. Garlic (*Allium sativum *L.) and onion (*Allium cepa *L.) are foods widely used in the entire world like spices. To date, many studies have reported the role of alliums in the prevention of several human diseases, including METS and cardiovascular disease, due to their effects on lowering lipids, blood pressure, and glycemia, as well as antioxidant effects [13, 14]. The major beneficial effects of alliums are attributable to the high content of organosulfur compounds produced when the garlic or onion tissue is damaged, and the odorless precursors are converted by the alliinase enzyme [13, 15]. The most important sulfur-containing compounds in onion bulbs are the amino acid cysteine and its derivatives. Onion also contains high levels of flavonoids, mainly quercetin as well as other phenolic compounds [16]. In contrast to onion, garlic mainly contains allicin derivative compounds such as ajoene, vinyldithiins, alkenyl-sulphides, depending on the type of solvent used. For example, aqueous garlic juice primarily contains alkenyl sulfides, such as diallyl disulfide and diallyl trisulfide [15, 17]. We recently demonstrated for the first time that aqueous garlic extract prevents vascular remodeling and oxidative stress in this experimental model of METS in relation to their organosulfur content [18]. In this study, we hypothesized that garlic and onion administration to FFR may reduce vascular inflammation related to their antioxidant properties. The main objective was to evaluate the effect of aqueous garlic and onion extract on VCAM-1 expression, aortic NAD(P)H oxidase, and heart eNOS activity in an experimental model of METS. Also, in order to evaluate the antioxidant capacity of alliums we used a control group treated with TEMPOL (4-Hydroxy-2,2,6,6-tetramethylpiperidine 1-oxyl, TEMPOL), a mimetic of superoxide dismutase enzyme [19].

## 2. Materials and Methods

### 2.1. Animals, Diet and Experimental Designs

All procedures were performed in accordance with institutional guidelines for animal experimentation. Thirty-day-old Wistar rats, weighing 95–120 g, were fed a standard commercial diet *ad libitum* and housed during the experimental period of 14 weeks in a room under conditions of controlled temperature (20°C), humidity, and a 12-hour light/dark cycle.

Administration of 10% fructose (Saporiti Labs., Buenos Aires, Argentina) solution as drinking water was used to achieve the pathological model (FFR). At the beginning of the study, fifty rats were randomly distributed in two groups for six weeks: one control group (C) (*n* = 10) and one experimental group FFR (F) (*n* = 40). After six weeks, the experimental group was further divided into four groups (*n* = 10 each) and for the subsequent eight weeks all groups received the following treatment: (i) control group (C); (ii) F; (iii) F + tempol (F+T); (iv) F + garlic (F+G); and (v) F + onion (F+O). All groups were fed the same standard rat diet (Gepsa-Feeds, Buenos Aires, Argentina) and tap water ad libitum. Standard diet containing (in g/kg) protein, 240; carbohydrates, 420; fat, 60; fiber, 70; vitamins and minerals. Aqueous garlic and onion extracts were administered orally in a dose of 150 mg/Kg/day and 400 mg/Kg/day, respectively. The doses were chosen based on previous studies [18, 20]. Tempol was administered in drinking water (1 mM).

Food intake and liquid consumption were recorded throughout the experiment.

At the end of the experimental period, and after overnight fast, the rats were weighed, anesthetized with ketamine (50 mg/kg) and acepromazine (1 mg/kg). Blood was collected from the abdominal aorta into heparinized tubes. Plasma obtained after centrifugation (1000 ×g, 15 min, 4°C, Sorvall RC-5B, SS-34 rotor, Wilmington, DE, USA) was frozen at −70°C until assayed. Arteries and organs from six rats of each group were excised aseptically for the measurement of various parameters described below. The remaining four rats of each group were designated for immunohistochemical analysis.

### 2.2. Garlic and Onion Preparations

Aqueous garlic and onion extracts (Fuego INTA and Refinta INTA, resp.) were obtained from the experimental station “La Consulta” of the National Institute of Agropecuary Technology (INTA), in Mendoza, Argentina. Thirty days after harvest, in appropriated store conditions, bulbs were peeled, weighed, and homogenized in distilled water, filtered, and diluted to obtain the required tissue concentration. Seventy five grams of garlic were diluted in 500 mL of water (final dilution was 150 mg/mL), and 200 g of onion were diluted in 500 mL (final dilution was 400 mg/mL). Extracts were aliquoted and stored (−70°C) until administered.

### 2.3. Biochemical Determinations

Plasma glucose and triglyceride concentrations were determined by enzymatic colorimetric methods using commercial kits (GTLab, Buenos Aires, Argentina). Insulin was measured by RIA (Coat-A-Count, Siemens, CA, USA), and insulin resistance was assessed using the homeostasis model assessment (HOMA-IR) with the following formula: HOMA-IR (mmol/L  ×  *μ*U/mL) = fasting glucose (mmol/L)  ×  fasting insulin (*μ*U/mL)/22.5. The SBP was monitored indirectly once a week in conscious, prewarmed, slightly restrained rats by the tail-cuff method and recorded on a Grass Model 7 polygraph (Grass Instruments Co., Quincy, MA, USA).

### 2.4. Markers of Oxidative Stress

The vascular NAD(P)H-oxidase activity in the aorta was measured by the lucigenin-derived chemiluminescence assay as previously described [7, 18], and the luminescence was measured on a luminometer (Fluoroskan Ascent FL; Thermo Labsystems, Waltham, MA). The results were expressed in arbitrary units (au)/min/mg of aortic tissue. 

Plasma thiobarbituric acid reactive substances (TBARS) concentration was determined according to previously described methods [5, 7, 18]. This method is based on the reaction between plasma malondialdehyde, a product of lipid peroxidation, and thiobarbituric acid, using as standard (1,1,3,3-tetramethoxypropane). The chromogen was spectrophotometrically measured at 532 nm (Shimadzu UV-160 Kyoto, Japan). The results were expressed in *μ*mol/L of TBARS, based on a standard curve with known quantities of malondialdehyde.

### 2.5. Measurement of eNOS Activity

The Ca^2+^/calmodulin-dependent nitric oxide synthase activity was measured in homogenates from left ventricular cardiac tissue by the conversion of L-[^3^H]arginine to L-[^3^H]citrulline, as previously described [5]. Heart tissue was homogenized on ice for four 15 s intervals with a polytron homogenizer and then sonicated in a buffer (pH 7.4, 37°C) containing 50 mmol/L Tris, 20 mmol/L HEPES, 250 mmol/L sucrose, 1 mmol/L dithiothreitol, 10 *μ*g/mL leupeptin, 10 *μ*g/mL soybean trypsin inhibitor, 5 *μ*g/mL aprotinin, and 0.1 mmol/L phenyl methyl sulphonyl fluoride. After centrifugation of the homogenates (100 g, 5 min, 4°C) and determination of the protein content (Bradford method), aliquots of 50 *μ*L were added to 100 *μ*L of a cocktail reaction buffer containing 50 mmol/L Tris, 20 mmol/L HEPES, 1 mmol/L dithiothreitol, 1 mmol/L NADPH, 0.1 mmol/L tetrahydrobiopterin, 50 *μ*mol/L FAD, 50 *μ*mol/L FMN, and 10 *μ*Ci/mL L-[2,3-^3^H]-arginine (New England Nuclear, Boston MA) and incubated for 30 min at 37°C in a shaking bath in the presence of 10 *μ*g/mL calmodulin and 3 mmol/L CaCl_2_ or with 3 mmol/L EGTA in absence of Ca^2+^/calmodulin. The reaction was stopped by adding 1 mL cold distilled water and the mixture was applied to an anion-exchange chromatography column containing Dowex AG 50W-X8 (200–400 Mesh) resin previously saturated with 50 *μ*L of 100 mmol/L L-citrullin and 2 mL of 50 mmol/L Tris, 20 mmol/L HEPES buffer (pH 7.4) and eluted with 2 mL of distilled water. Specifically eluted L-[^3^H]citrulline concentration was determined by liquid scintillation counting. The calcium-dependent NOS activity was calculated as the difference between activity in the presence and absence of Ca^2+^/calmodulin. Values were corrected according to the amount of protein present in the homogenates and the incubation time (dpm/mg protein/min). Rat heart tissues were processed and eNOS activity measured independently.

### 2.6. Protein Extraction and Western Blot Analysis

Vascular mesenteric tissues were dissected, weighed, and immediately stored at −70°C until analysis as previously described [7]. Briefly, 100 mg of each sample was homogenized on ice in 500 *μ*L lysis buffer pH 7.4 containing 10.9% sucrose, 0.037% EDTANa_2_, 0.06% HEPES, and proteinase inhibitors (100 *μ*g/mL of phenylmethanesulfonyl fluoride and 1 *μ*g/mL soybean trypsin inhibitor). Protein concentrations were quantified in duplicate using Bradford method with bovine serum protein as reference protein. 

Twenty micrograms of total protein were separated in SDS-PAGE 8% and transferred onto nitrocellulose membranes (Amersham Biosc., Buckinghamshire, UK). Molecular weight markers were run in parallel. Membranes were incubated overnight at 4°C with primary antibody, anti-VCAM-1 (rabbit polyclonal, 1/500, Santa Cruz Biotechnology Inc., CA, USA) in PBS-T solution. Monoclonal anti-*β*-actin antibody (1/4000) was used as control for equal loading. Horseradish peroxidase-conjugated anti-rabbit and anti-mouse secondary antibodies (1/2500 and 1/4000, resp., Dako, Glostrup, Denmark) were used, and immunoreaction signals were viewed with enhanced chemiluminescence (Hyperfilm ECL, Amersham Biosc., Buckinghamshire, UK). X-ray films with protein bands were scanned and densitometric analysis was performed using the US National Institute of Health Image 1.66 software (Rasband Wayner et al. Division of Computer Research and Technology NIH, Bethesda, Maryland, USA). Data was expressed in arbitrary units as primary antibody/*β*-actin ratio.

### 2.7. Immunohistochemistry Analysis

To assess VCAM-1 protein location in endothelial cells of mesenteric tissue, four animals of each group were perfused as previously described [10] and mesenteric tissue was embedded in paraffin. Slices were cut transversely on a microtome (Microm HM325, Walldorf, Germany) with a thickness of 5 *μ*m each, and dewaxed in xylol, rehydrated, and incubated with 1.5% hydrogen peroxide (H_2_O_2_) for 30 min to quench endogenous peroxidase activity. After washing in tris-buffered saline 0.05% Triton X-100 (0.05 M Tris, 0.15 M NaCl; pH 7.6), and nonspecific blocking with 1% BSA, nonfat milk (3.5%) for 30 min at room temperature, the tissue was incubated overnight with primary antibody anti VCAM-1 rabbit polyclonal (dilution 1/50), from Santa Cruz Biotechnology, Santa Cruz, CA, at room temperature. After additional washings, sections were incubated with a biotinylated-secondary anti-rabbit antibody (dilution 1/100) during 2 hours at room temperature and then incubated with extravidin-HRP (dilution 1/100) for 1 hour. Finally, the sections were immunostained using diaminobenzidine, glucose oxidase plus nickel ammonium sulfate (as color enhancer) was used as chromogen. Slices were mounted, dehydrated, and coverslipped with synthetic Canadabalsam (Laboratorio Cicarrelli, Buenos Aires, Argentina). The images were examined under an optic microscope (Nikon Optiphot-2, Kanagawa, Japan) and digitalized with a digital camera (Panasonic GP-KR222 color CCD, Panasonic, Osaka, Japan).

### 2.8. Reagents

Unless otherwise noted, all reagents were purchased from Sigma Aldrich (St. Louis, MO, USA). All other chemicals were of molecular biology or reagent grade.

### 2.9. Statistical and Data Analysis

Data were expressed as mean ± SEM. The statistical significance was assessed by one-way ANOVA followed by Bonferroni's Multiple Comparison posttest. GraphPad Prism version 5.00 for Windows (GraphPad Software, San Diego, CA, USA) was used for all statistical analyses. Differences were considered significant at *P* < 0.05.

## 3. Results

Fructose solution intake was higher in F, F+G, F+O, and F+T group than water consumption by C group (*P* < 0.01). All experimental groups ate less food than C group (*P* < 0.05). The addition of garlic, onion, or tempol to F rats did not modify the food or liquid intake. Although the energy intake was higher in F, F+T, F+G, and F+O groups than in the C group (*P* < 0.05), we did not find significant differences in body weight among groups (Table 1). 

At the end of the experimental study FFR showed a significant increase in SBP, fasting levels of plasma glucose, insulin, insulin resistance index, and triglycerides (*P* < 0.01) compared to control group. FFR receiving tempol, garlic, and onion showed a decreased SBP (*P* < 0.05) similar to the C group, slightly reduced insulin levels, but did not show modified levels of glycemia and triglyceridemia. The HOMA-IR index was significantly reduced when garlic and onion were administered to FFR, but did not reach control levels (Table 2). 

**Figure 1:**
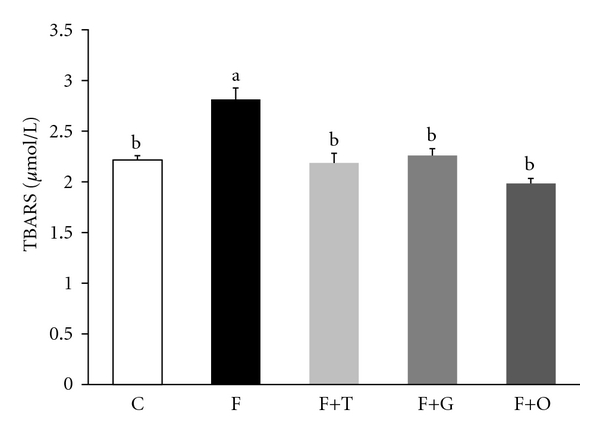
Plasma TBARS from C, F, F+T, F+G, and F+O groups. Values are expressed as mean ± SEM, (*n* = 6); bars with different superscripts differ, *P* < 0.05.

**Figure 2:**
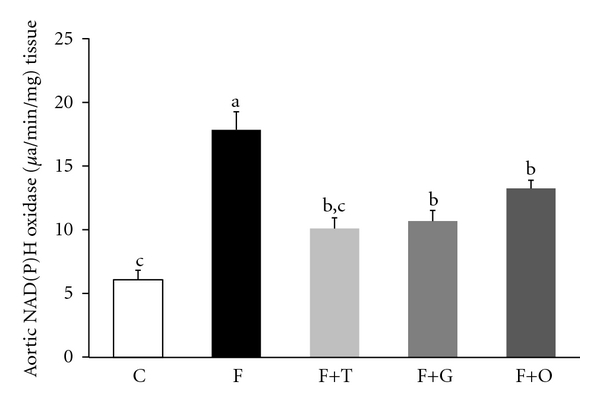
Aortic NAD(P)H oxidase activity C, F, F+T, F+G, and F+O groups (*n* = 6 rats per groups). Values are expressed as mean ± SEM, (*n* = 6); bars with different superscripts differ, *P* < 0.05.

When the oxidative parameters were evaluated, the plasma levels of lipid-peroxidation and the NAD(P)H oxidase activity in aortic tissue was significantly higher in the F group compared to the C group (*P* < 0.01) (Figures 1 and 2, resp.). Administration of tempol, garlic, and onion to FFR significantly reversed the levels of lipid-peroxidation and NAD(P)H oxidase activity (*P* < 0.05).

**Figure 3:**
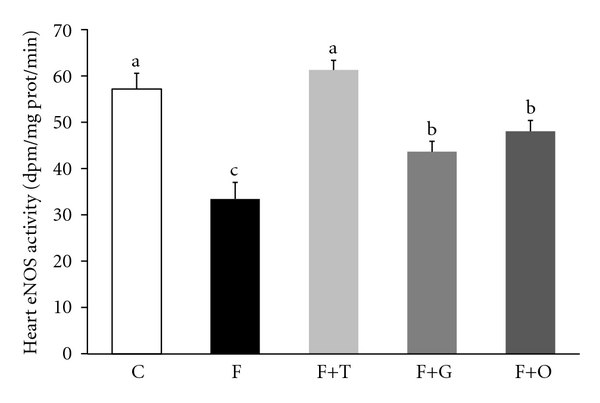
Heart endothelial nitric oxide synthase (eNOS) activity from C, F, F+T, F+G, and F+O groups. Values are expressed as mean ± SEM, (*n* = 6); bars with different superscripts differ, *P* < 0.05.

The eNOS activity measured in heart tissue from left ventricle homogenates decreased significantly in the F group, compared to control rats (*P* < 0.01). Long-term treatment with tempol, garlic, and onion was able to partially avoid the reduction of eNOS activity in the FFR left ventricle homogenates (*P* < 0.05 versus FFR), while tempol administration increased the enzyme activity to levels close to the control group (Figure 3).

**Figure 4:**
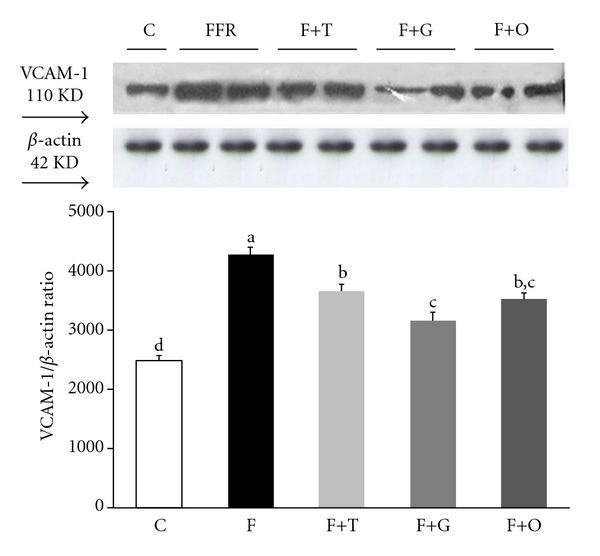
Vascular cell adhesion molecule-1 (VCAM-1) protein expression in vascular mesenteric tissue assessed by western blotting from C, F, F+T, F+G, and F+O groups. Values are expressed as mean ± SEM, (*n* = 4); bars with different superscripts differ, *P* < 0.05.

**Figure 5:**
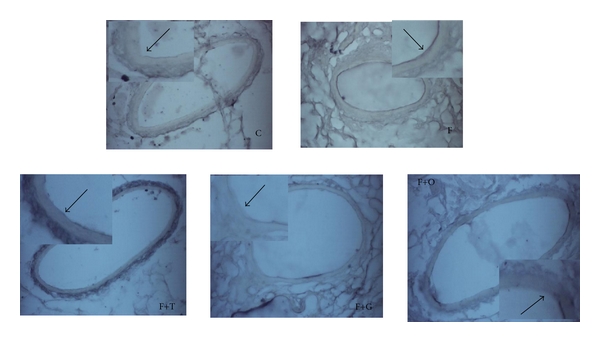
Immunohistochemistry analysis of VCAM-1 in endothelial cells of vascular mesenteric tissue from C, F, F+T, F+G, and F+O groups (*n* = 4). Each photo shows an enlarged image. The arrows indicate the VCAM-1 expression in the endothelium: magnification ×400.

The vascular inflammation was assessed by western blot by measuring VCAM-1 in mesenteric vascular tissue. FFR showed a significant increase in VCAM-1 protein expression compared with C group (*P* < 0.05). Tempol, garlic, and onion treatment administered to FFR induced a significant reduction of VCAM-1 expression. The western blot assay was corroborated by the immunohistochemistry analysis of VCAM-1 in mesenteric endothelial cells (Figures 4 and 5, resp.).

## 4. Discussion

In the present study, we show that aqueous fresh garlic and onion extracts have the ability to reverse (i) the increase of SBP, (ii) oxidative stress, (iii) the reduction of heart eNOS activity, and (iv) the increase of vascular inflammation in FFR.

According to previous studies [5, 7, 10, 18], fructose-fed animals develop insulin resistance, hypertriglyceridemia, increased SBP, oxidative stress, and vascular remodeling. Aqueous garlic and onion administration to FFR for 8 weeks was able to reverse the increased SPB and reduce the insulin resistance index, but did not significantly modify the plasma levels of glycemia, insulinemia, and triglyceridemia. These results are consistent with those found in a previous study where we evaluate the effect of two garlic cultivars in FFR [18].

Several studies have shown the beneficial effects of garlic and onion in reducing SBP, mainly attributed to their antioxidant effects [21]. Some of the mechanisms of alliums involved in lowering blood pressure are related with an increase of NO bioavailability, a strong vasodilator [13, 22]. Other authors postulate that alliums increase eNOS activity, thereby increasing NO production [23, 24]. *In vitro*, the gamma-glutamyl-cysteines present in garlic lowered the SBP by inhibiting the angiotensin-converting enzyme [25]. In accordance with these findings, we found that garlic and onion increase the heart eNOS activity in FFR. Furthermore, we also found that both garlic and onion were able to reduce the activity of aortic NAD(P)H oxidase and lipid-peroxidation levels in plasma, supporting the hypothesis that these vegetables may reduce SBP by increasing NO bioavailability. Garlic and onion are vegetables present in the Mediterranean diet, which is characterized by its antioxidant properties mainly due to the high organosulfur content [15]. In vascular tissue, the membrane-associated NAD(P)H oxidase accounts for the majority of superoxide generation favoring the production of ROS [8, 26], which has an important role in vascular inflammation [27]. Excessive ROS production may underlie pathologic processes associated with endothelial dysfunction and vascular remodeling, which are characteristic features in hypertension [28]. Accumulating evidence suggests that ROS control vascular gene expression through specific redox-sensitive signal transduction pathways [27]. Nuclear factor kappa B activation participates in the transcription of numerous proinflammatory genes, including vascular cells adhesion molecules, and it is one of the transcription factors influenced by cellular redox state [29]. VCAM-1 mediates, primarily, the adhesion of monocytes, lymphocytes, eosinophils, and basophils to the surface of endothelial cells, promoting migration through the endothelium [30]. In this study, the increased oxidative stress in FFR was related with an increase of VCAM-1 expression in vascular mesenteric tissue assessed by western blot and immunohistochemistry. Long-term administration of garlic, onion, and tempol was able to attenuate the VCAM-1 expression in FFR. The effect of some organosulfur compounds derived from garlic on vascular cell adhesion has been studied in human endothelial cells [31]. Son et al. [32] reported that allicin inhibits intracellular cell adhesion molecules in human vascular endothelial cells via downregulation of the c-Jun N-terminal Kinase signaling pathway. Contrarily, Ide and Lau [33] showed *in vitro* that aged garlic extract and one of its major compounds, S-allylcysteine, protect endothelial cells from oxidized-LDL-induced injury by preventing the depletion of intracellular glutathione and by removing peroxides. S-allylcysteine also inhibited the nuclear factor kappa B activated by TNF-alpha or hydrogen peroxide. In spite of these reports, studies of onion's effect on vascular inflammation are less advanced. Notwithstanding, recent studies have been conducted regarding the anti-inflammatory effect of quercetin, the main flavonoid in onion [34, 35]. In obese Zucker rats, high doses of quercetin attenuates the metabolic syndrome and improves the inflammatory status in visceral adipose tissue evidenced by a reduction in TNF-alpha production, decreased fat iNOS expression, and an improved eNOS expression [36].

## 5. Conclusions

In conclusion, our findings suggest that the ability of garlic and onions to act as antioxidants, decreasing NAD(P)H oxidase activity and lipid-peroxidation, increasing the activity of eNOS, and attenuating the vascular inflammation in FFR as evidenced by a decrease in VCAM-1 protein expression, providing new evidence showing the anti-inflammatory effect of these vegetables. These results highlight the importance of the consumption of natural vegetables and also contribute to the understanding of the beneficial effects of functional foods in the prevention of METS.

## Figures and Tables

**Table 1:** Fluid, food, energy intake, and body weight from C, F, F+T, F+G, and F+O groups.

Variables	Groups				
	C	F	F+T	F+G	F+O
Liquid intake (mL/day)	25.2 ± 1.2^b^	35.7 ± 2.1^a^	35.3 ± 2.2^a^	35.5 ± 2.1^a^	34.9 ± 2.3^a^
Food intake (g)	18.4 ± 0.6^a^	16.2 ± 0.4^b^	16 ± 0.4^b^	15.7 ± 0.6^b^	16.1 ± 0.7^b^
Energy intake (KJ)	245 ± 8^b^	275 ± 11^a^	272 ± 9^a^	268 ± 11^a^	273 ± 13^a^
Body weight (g)	320 ± 7	341 ± 8	331 ± 7	328 ± 9	327 ± 8

Values are expressed as mean ± SEM, *n* = 10 for each group; means in a row with different superscripts differ, *P* < 0.05.

**Table 2:** Systolic blood pressure (SBP) and metabolic variables from C, F, F+T, F+G, and F+O groups.

Variables	Groups				
	C	F	F+T	F+G	F+O
SBP (mmHg)*	122.6 ± 2.1^b^	132.6 ± 2.4^a^	119.4 ± 1.8^b^	120.7 ± 1.7^b^	122.5 ± 1.3^b^
Plasma glycemia (mmol/L)	4.7 ± 0.5^b^	6.8 ± 0.7^a^	6.4 ± 0.6^a^	6.5 ± 0.5^a^	6.3 ± 0.4^a^
Plasma insulin (pmol/L)	81.3 ± 8.4^b^	160.4 ± 9.0^a^	138.9 ± 8.3^a^	133.3 ± 9.4^a^	130.3 ± 8.2^a^
HOMA:IR	2.4 ± 0.4^d^	7.0 ± 0.4^a^	5.7 ± 0.3^ab^	5.5 ± 0.3^bc^	5.3 ± 0.3^bc^
Plasma triglycerides (mmol/L)	0.72 ± 0.09^b^	1.12 ± 0.07^a^	1.01 ± 0.06^a^	0.98 ± 0.08^a^	1.01 ± 0.07^a^

Values are expressed as mean ± SEM, *n* = 6 in all variables except *n = 10; means in a row with different superscripts differ, *P* < 0.05.
